# *“We have to change our mindsets”*: a qualitative study of barriers and facilitators in research collaboration across integrated care system organisations

**DOI:** 10.1186/s12913-024-10760-3

**Published:** 2024-03-01

**Authors:** Christopher J. Gidlow, Lorna Sams, Kim Buckless, Naomi J. Ellis, Helen C. Duffy, Ruth Lambley-Burke, Paul Campbell, Alison Cooke, Krysia Dziedzic, Matthew Brookes, Nachiappan Chockalingam, Pam Devall, Christian Mallen

**Affiliations:** 1 Centre for Health and Development (CHAD), Staffordshire University, Ashley Building, Leek Road, ST4 2DF Stoke-on-Trent, UK; 2 Midlands Partnership University NHS Foundation Trust, St George’s Hospital, Corporation Street, ST16 3AG Stafford, UK; 3 Keele University, ST5 5BG Keele, Staffordshire, UK; 4 University Hospitals of North Midlands NHS Trust, Newcastle Road, ST4 6QG Stoke-on-Trent, UK; 5 University of Wolverhampton, Wolverhampton, UK; 6 Department of Gastroenterology, New Cross Hospital, Wolverhampton, UK; 7 NIHR Clinical Research Network West Midlands, New Cross Hospital, WV10 0QP Wolverhampton, UK

**Keywords:** Integrated care, Collaboration, Qualitative

## Abstract

The introduction of Integrated Care Systems (ICS) in England aimed to increase joint planning and delivery of health and social care, and other services, to better meet the needs of local communities. There is an associated duty to undertake collaborative research across ICS partners to inform this new integrated approach, which might be challenging given that organisations span health, local authority, voluntary and community sector, and research. This study aimed to explore the appetite for collaborative Research and Innovation (R&I) across ICSs, potential barriers and solutions. This qualitative study involved semi-structured interviews with 24 stakeholders who held senior positions within organisations across two ICS areas (Staffordshire and Stoke-on-Trent; Shropshire, Telford and Wrekin). Interview transcripts were analysed using inductive and deductive analysis, first mapping to the Theoretical Domains Framework (TDF), then considering key influences on organisational behaviour in terms of Capability, Opportunity and Motivation from the COM-B Behaviour Change Wheel. There were fundamental limitations on organisational *opportunities* for collaborative R&I: a historical culture of competition (rather than collaboration), a lack of research culture and prioritisation, compounded by a challenging adverse economic environment. However, organisations were *motivated* to undertake collaborative R&I. They recognised the potential benefits (e.g., skill-sharing, staff development, attracting large studies and funding), the need for collaborative research that mirrors integrated care, and subsequent benefits for care recipients. Related barriers included negative experiences of collaboration, fear of failing and low confidence. *Capability* varied across organisations in terms of research skills and confidence, which reflected the range of partners (from local authorities to NHS Trusts, primary care, and academic institutions). These findings indicate a need to shift from a culture of competition to collaboration, and to help organisations across ICS to prioritise research, and share resources and skills to mitigate the limiting effects of a constrained economic environment. This could be further explored using a systems change approach, to develop the collaborative research efforts alongside the overarching move towards integrated care.

**Supplementary Information:**

The online version contains supplementary material available at 10.1186/s12913-024-10760-3.

## Introduction

Since July 2022, local health and social care in England has been mandated to plan and deliver services through Integrated Care Systems (ICS). They are described in the NHS Long Term Plan [1] as part of the solution to address the wider determinants of health by increasing the number of health and non-health services that can be provided jointly, and give an infrastructure to enable NHS organisations to work with local partners to meet the needs of their communities.

NHS England also stated that the inception of ICSs ‘*provides the opportunity for systems to embed research within health and care for the benefit of our population*’ [2]. The potential benefits of collaboration between partners involved in policy, practice and research are widely recognised: improving public health and care provision (and the associated evidence), fostering multi-disciplinary working and innovation, and the sharing of knowledge and risk [3–5]. Such research–practice collaborations are thought necessary to address the complex, substantial public health challenges [5, 6].

Whilst endorsement of research and evidence-based/informed practice is encouraging, there are important considerations. First, partnership working is not easy. In the UK Health and Social Care context [7], recent appraisal highlighted the challenges from uneven funding across sectors (particularly non-health based organisations), and the need to foster greater collective focus on how ICS success can be measured at a local level [8]. Second, and perhaps of greater importance in the establishment of ICS research, is the need for appropriate research infrastructure. ICSs cover a range of organisation types and sectors, from NHS to local authorities, voluntary and community organisations, and Universities, which are likely to differ in their research experience, culture and practices. To help build the necessary infrastructure, the Health and Care Act 2022 [9] formalised the statutory components in the form of the Integrated Care Boards (ICBs, responsible for planning and fundings NHS services), with duties that include facilitating or promoting research relevant to the health and care services, and its use in health service delivery [9]. Fulfilment of these statutory duties will require a genuine commitment to collaborative research across ICS organisations (some of which might not have previously engaged), which should be in line with the developing ICS research strategies [10].

This qualitative study took place in the North West Midlands of England, an area with long-standing partnership arrangements between health care organisations and academic institutions, and associated research activity (e.g., NHS Primary Care Research Consortium). Here, ICS partners recognised that NHS reorganisations can pose a serious impediment to research. NHS re-structuring inevitably leads to management of change processes, changes in personnel and organisational structures that can be difficult for researchers and clinicians to navigate [11, 12]. For example, the creation of Primary Care Trust’s (in 2001) and subsequent creation of Clinical Commissioning Groups (in 2013) presented both opportunities (in research being seen as important beyond ‘traditional NHS Trust’ boundaries) and challenges (in working out who was responsible for what and how funding and costs should be managed), with research responsibilities not clearly defined.

The inception of ICS has meant a greater number and diversity of partner organisations involved in care delivery and associated research. This has increased the potential for, and challenges of, collaborative research and innovation (R&I). Therefore, in 2019, the Staffordshire and Shropshire Health and Care Research Partnership (SSHERPa) was established to enhance opportunities for collaborative research and impact across two ICS areas (NHS Staffordshire and Stoke-on-Trent and NHS Shropshire Telford and Wrekin) in the North Midlands. SSHERPa brought together local authorities, NHS organisations (at the time NHS Trusts/CCGs), Higher Education Institutions and NIHR infrastructure (CRN WM). Critically, SSHERPa aimed to ensure that, amidst the change and disruption of ICS development, existing relationships and partnerships were not lost, that new partnerships were developed and supported, and that barriers to R&I could be identified and addressed.

This study was undertaken to explore the appetite for collaborative R&I, to understand the potential barriers, and possible solutions of collaborative research across a range of health and care organisations.

## Methods

### Study design

This qualitative study involved semi-structured interviews with ICS stakeholders who were decision-makers within their organisations, drawing on the Theoretical Domains Framework (TDF) [13] and COM-B Behaviour Change Wheel [14]. The TDF was developed to simplify and integrate psychological and organisational theories relevant to health practitioner behaviour change [13]. It comprises 14 domains of factors that influence behaviour (e.g., knowledge; skills; social/professional role and identity; beliefs about capabilities; beliefs about consequences; motivation and goals; environmental context and resources; social influences). The TDF has been used in healthcare systems to explain implementation problems (e.g., [15–17]) and to inform interventions (e.g., [18]). It also maps to the well-established COM-B [14]. TheCOM-B model was developed to understand the conditions required for a specified behaviour to occur, organising them into broad categories of Capability, Opportunity, and Motivation. These, in turn, map to intervention functions and types of policy to deliver them [19]. Similar to the TDF, the COM-B was developed in the context of individual behaviour change, but considers influences at a range of levels, and has been used to examine determinants of cooperation between organisations and sectors in other contexts [20].

In the present context, the TDF was used to identify influences on collaborative research based on data collected at the individual level, from decision makers able to affect the behaviour of their organisation (and with relevant knowledge of organisational R&I activities). The types of influences identified included individual and organisational factors, in addition to influences of the wider system (e.g., ICS, economic conditions). The TDF is most used to examine determinants of individual-level behaviours, often health professionals, but has been used to consider influences at individual, organisational and system levels [21]. Ethical approval was obtained from the Staffordshire University Ethics Committee (REF: SU_22_059).

### Participants

Participants were a purposive sample of stakeholders with key positions in the ICS. Types of organisations included NHS Trusts, local authorities, National Institute for Health and Care Research Clinical Research Network, Academic Health Science Networks, Primary and Secondary Care, third sector partners, and Universities. The type of stakeholder within those organisations included Research and Development managers and executive-level leads; decision makers with knowledge of relevant organisational influences and practices, whose individual behaviours could affect the behaviour or their organisation (regarding R&I). There were no specific exclusion criteria.

### Study procedures

#### Recruitment

A list of potential participants was collated by the SSHERPa group and reflected the membership of SSHERPa at that time (in 2022). Identified individuals were sent an introductory email by the SSHERPa programme manager, and follow-up email with an information sheet and consent form from the research team (up to two reminders). Additional calls to provide further detail were arranged as appropriate. For those willing to participate, an interview was arranged at a mutually convenient time. Participants were then asked to complete and return the consent form and sent a list of the topics that would be covered during the interview, to allow them time to consider their responses. This was important in cases where individuals had not previously given consideration to R&I.

#### Data collection

Semi-structured interviews were carried out by author LS over the telephone or online (MS Teams) from November 2022 to January 2023. LS is a female researcher with extensive experience of qualitative research in health care settings, but no prior knowledge of, or relationship with any participants. Only the interviewer and participant were present during interviews. An interview topic guide was developed for this study in partnership with stakeholders from the SSHERPa network and informed to some extent by the TDF (whereby questions were mapped to domains to determine potential to elicit response relating to different parts of the TDF; see Supplementary file 1). Interviews were audio-recorded and transcribed verbatim (by a professional transcriber) for analysis.

#### Data analysis

Data analysis involved inductive then deductive phases, an approach employed elsewhere to draw on the TDF and COM-B [22, 23]. Use of implementation frameworks and the deductive processes align the analysis with positivist or realist positions [24], although the initial inductive phase was included to ensure that issues that might fall outside the TDF would not be excluded. First, two experienced qualitative researchers (LS, KB) independently coded transcripts (KB had expertise in health psychology). These were checked for alignment with the TDF domains in discussion with a third researcher (NE, a senior qualitative researcher). The codes did align with eight of the 14 TDF domains, in some cases with an organisational, rather than individual focus. For example: findings under Skills were more relevant to skills across the organisation, not just those of the interviewee; findings under Social/Professional Role and Identity were more relevant to constructs such as group identity, leadership, organisational commitment, rather than the individual-level constructs [13]. Therefore, the TDF domains were not amended prior to the second phase in which the remaining transcripts were deductively coded using the TDF constructs/domains. Third, the results were reviewed with another researcher (CG, a senior researcher with experience of using TDF and COM-B) and, where concepts were relevant to more than one TDF construct, the most appropriate position was agreed, to avoid repetition. Finally, findings for each domain were consolidated (rather than reporting by construct) and then considered in the context of the COM-B. All analysis was undertaken in NVivo R1.

## Results

Twenty-five interviews were conducted by MS Teams (*n* = 24) or telephone (*n* = 1), lasting an average of 28 min (range 16–60 min). This represented 68% of the 37 invited (with the remainder not responding to invitations). We interviewed all of the potential population of stakeholders that responded to invitations, but were confident that no new themes were emerging by the end of data collection. Stakeholders’ provided descriptions of their roles regarding research which ranged from responsibility for strategy, to research delivery, management of R&I, dissemination and seeking external funding. Findings are presented in relation to the COM-B and TDF domains and with illustrative quotations denoted by anonymised participant numbers (e.g., P1, P2, etc.). No further individual participant information is given to protect their identities. Table 1 summarises main themes and which level of influence they relate to (individual, organisational, wider system), which are described in turn.

**Table 1:** Summary of main themes by level, mapped to theoretical domains framework (13) and COM-B (14)

COM-B component	TDF Domain*	Summary of main themes		
		Individual level	Organisational level	Wider/system level
Capability	Knowledge	• Variation in stakeholder knowledge of processes for collaborative R&I	• Variation in whether organisations have processes for collaborative R&I • Processes are clearer for research than innovation • Need for collaborating organisations to be protected through formal agreements, and data sharing agreements	Knowledge of processes for R&I made difficult as ICB and ICS development has meant that such processes are in flux
	Skills	• Communication and relationship building skills are necessary to bring multidisciplinary teams together for collaborative R&I • Research skills vary within organisations	• Communication and relationship building skills are necessary to bring multidisciplinary teams together for collaborative R&I • Research skills vary across organisations • Collaboration is beneficial for research skill-sharing	• Need better awareness of where research expertise is across the ICS partner organisations
Opportunity	Environmental Context and Resources		• COVID-19 - Increases in remote working increases have negatively impacted opportunities to collaborate - COVID-19 R&I (e.g., vaccine development) showed that collaborative, dynamic R&I is possible when traditional barriers are removed - Changes in research focus led to new partnerships • Importance of organisational culture to ensure that R&I is prioritised • Traditional competition around research remains dominant, highlighting a need to shift from competition to collaboration	• COVID-19 impact on the system • Pressured economic environment
Motivation	Social/ Professional Role and Identity	• Individuals do not always feel supported to encouraged to undertake joint research	• Organisations at different stages of development for research • Organisational commitment is required at board and senior leadership levels • True collaboration involved a collective responsibility and shared risk	• Need to harmonise research across participating organisations, while recognising institutional targets
	Beliefs about Capabilities		• Variation in organisational confidence for R&I • A fear of failing prevents innovation • Track record and credibility for research fosters collaboration	
	Beliefs about Consequences		• Belief that collaboration is beneficial (skill-sharing, collective strength) • Imbalanced collaborations can be detrimental: - Small partners not being heard / larger organisations dominating - Larger organisations assume more workload than planned (if there is lack of engagement from smaller partners) - Unequitable allocation of finances • Need agreements around workload and have common aims	• Belief that collaboration is necessary: - to cover the R&I needs of a large and diverse ICS geography - as services are integrating across partner organisations, so must R&I
	Goals	• Individuals lack time for innovative activities to facilitate goal of increasing collaborative research	• To build R&I representation at organisational board-level • To undertake more collaborative research	• Specific actions to achieve goals include: - A shared framework for R&I across ICS partner organisations - Having a shared directory - Running webinars and events to foster partnership - Having have shared roles to increase capacity and address skill needs
	Optimism	• Pessimism about lack of capacity for research when it is not a core part of individual’s job roles	• Optimism regarding the benefits of collaborative R&I through: - Shared risks - Stronger study teams • Pessimism about: - The integrity of collaborations and trust for sharing of ideas (i.e., competitive culture remains)	• Optimism about a prominent role for R&I in the ICS • Optimism regarding the benefits of collaborative R&I through: - Attracting larger studies to the region - Collective expertise and strength - Allowing higher quality research and more innovation - Lead to better services/care and patient outcomes

*Domains not included as not relevant to the data: Reinforcement; Intentions; Memory, Attention and Decision Processes; Social influences; Emotion; Behavioural Regulation

### Capability

#### TDF domain: knowledge

Knowledge of how to develop and deliver R&I reflected a mixed and changing picture. Some stakeholders were unaware of processes within their organisation for R&I: “we don’t actually have anything at the moment… it is more an…as we go along [process]” (P23). Others had knowledge of the processes for conducting research, but less so for innovation: “our processes in…[innovation] are way behind our processes in research in terms of being tight, well managed, well-rehearsed, and practical” (P20).

There was a perception that the development of ICSs meant “a lot of the processes and new pathways are in this state of flux” (P9). Although stakeholders were aware of “robust governance structures” (P4) within their organisations, processes for collaboration could be “quite ad hoc” (P13). They expressed a need for “overarching agreements…to support research, delivery capacity” (P3) and when “there’s any delegation of responsibility or any funds going either way, [then] we will need contractual arrangements in place, so that’s when procedures can kick in” (P7). Stakeholders were also keen to have “data sharing agreements…[to] ensure that organisations are protected” (P5). Issues with data sharing had caused delays or partners withdrawing: “we’ve tried to pull datasets across organisations…but the legislation and breaking down the data protection barriers [has]…massively stalled [projects]” (P13). There was also evidence that different practices and IT systems across organisations could limit opportunities for collaboration: “we don’t have the same digital technology which talks to each other…which might delay things to be working collaboratively…even if it’s across local authority and different organisations” (P27); “if we don’t have shared IT systems and good communications, then we’re not building…[collaboration] on a good foundation” (P39).

#### TDF domain: skills

Related to skills, stakeholders spoke about the importance of interpersonal skills and communication, the benefit of collaboration for sharing of research skills across organisations, and how this might address some of the research skill gaps.

At the individual level, effective communication and relationship building skills were commonly cited requirements for successful collaboration. Others noted their organisational advantage of being a “multi-disciplinary, multi-agency player [to]…bring people together and collaborate” (P33), “inside…and outside the region” (P4). A potential weakness was that collaboration was underpinned by “individuals…who are passionate and driven to develop research” (P35). This reflected dependence on individual relationships can mean that if project leads “move out of the area, they can often take that [project] with them and then it doesn’t continue in the same collaborative way” (P10).

There was a perceived need to develop research skills in some partner organisations. At the individual level, some stakeholders reported having a “strong grounding in research skills” (P4) and being able “to set up and deliver the research” (P29). Others referred to organisational “gaps in…general research skills” (P13). Part of the solution identified was specific training for “skills development with some of the officer level posts” (P13), or “creative shadowing opportunities” (P23). Upskilling of staff was also considered beneficial to prevent them “being poached by another organisation” (P23).

Collaboration was thought valuable through bringing together people with skill sets that are “complementary…you can pull in different strengths…expertise…[and] viewpoints” (P4) or working with “organisations that were big on providing training which could be extended out as a collaborative” (P5). For this to happen, greater knowledge sharing and communication across ICS organisations was necessary, otherwise “there’s a danger you could always end up collaborating with the same people, because they’re the people that you know, instead of the best -person who might be based somewhere else, but you just didn’t know who they were” (P17).

*in order for us to work more collaboratively on things on a bigger scale, we need to understand each other’s organisations and remits a bit better…[it] can sometimes create a little bit of friction when we don’t maybe fully understand each other’s roles *(P39).

### Opportunity

#### TDF domain: environmental context and resources

Stakeholders identified several ways in which organisational and wider environmental context and resources influenced their opportunities for collaborative R&I, many of which were discussed as barriers.

The change in working practices as a result of COVID-19 have negatively impacted opportunities to develop interpersonal skills and relationships:

*Since COVID, a lot more people are working from home and…offices aren’t being utilised…I don’t think we’ll ever be in that situation again where we can all work under the same roof, all at the same time where you can have…conversations* (P39).

Whilst there was a perception that the COVID-19 response had demonstrated how rapid, collaborative research was possible, it had also altered the focus in some organisations. For example, shifting the focus to “public health type research, which has taken us away to some extent away from a lot of the clinical, academic and commercial research that we might have focused on otherwise” (P32). Such changes had led some organisations to build new relationships, again, reflecting the changes as ICSs develop: “the health and care landscape is changing, and we’ve got new stakeholders…that we’ve probably not worked with before” (P5).

Stakeholders frequently discussed the importance of organisational culture around research: “culturally, we’ve got to be very clear research isn’t…a luxurious add on” (P33). Changes related to ICS development were discussed in terms of organisations becoming “very much…collaborative” (P10) and “committed to research and innovation” (P32). One stakeholder stated that “our main thrust now is to work with the structures within these new organisations that form ICS to enable us to become research active” (P35). But this commitment, both in principle and in resources, was variable. For example, in one organisation, “research, development, innovation [was] part of their day job, and not in addition” (P33). Others commented that support services were “too busy…doing the clinical work… capacity to effectively free up resource to do research is severely constrained” (P22).

*we all struggle to find time in the day [for research]…Some organisations offer protected time…but often we give that up because something else…[takes] precedence, and then sometimes we can be a bit resentful that we’ve got to do that in our own time* (P39).

This reflected a combination economic pressures compounding the challenges for organisations in which research is not embedded: “we probably don’t do anywhere near as much [research] as we could, because…of…political challenges and resource challenges” (P16). This was specifically noted as a barrier to collaborative research funding proposals, which can be more time consuming:

*Bids for external research funding are always very last minute…and doing a collaborative bid in that time…can be really tricky…[and is] sometimes off putting and limiting, particularly if it’s not even a very big pot of money…and you have to then balance the benefit of that with the time that goes into it (P13)*.

There was a perceived need for greater “investment and…a slight change of mindset” (P22) in some organisations, particularly to foster collaborative R&I. Stakeholders reported that they are “all working in silos…we probably all need to work together as single system organisations and then bond well from our own strengths [and] support each other…in terms of conducting research” (P18). For example, local authorities were noted to “have certain ways of doing things…lots of statutory duties, lots of roles and responsibilities and a lot of those aren’t conducive to innovation” (P16). Ultimately, differing organisational priorities and agendas remain a barrier:

*competing priorities and agendas will always make [collaboration] tricky…if you’re not all reporting to the same Senior Leadership Team, and you’re not all working to the same budget. Any collaboration…comes with…challenges around getting everyone to commit, getting everyone on the same page [and] the various reporting structures (P13)*.

Part of the required cultural change thought necessary to increase research opportunities was a shift from competition to collaboration. Some stakeholders spoke of a “culture…that we keep things within us, in our bubble… We collaborate, but only…with those people that we want to” (P21). Stakeholders described organisations as being “a little territorial” (P3) and unwilling to share information and ideas: “we’re not encouraged…to share work at all, not in this healthy economy, it’s every Trust for themselves” (P26).

*You’re fighting against each other…for the funding and it is changing the way we think…to making it a collaboration where we can work together to get the funding, instead of trying to do things on our own…we have to change our mindsets and that’s difficult to do, because historically, it’s always been very competitive (P17)*.

Previous collaborations were described as a “learning process” and the “bedrock for ongoing work” (P6). In addition, collaborations have supported “other organisations that don’t have that infrastructure to develop their own…research” (P3) and it made “sense for there to be some synergy in terms of working, rather than both organisations going off in different directions, actually having some sense of cohesion was more efficient” (P4).

### Motivation

#### TDF domain: Social/professional role and identity

Roles and identities were largely discussed at the organisational level. Roles relating to research were varied, with some organisations still “developing” (P39) and not yet “as active as we should be in research” (P32). Linked with organisational culture, individual stakeholders said they were “not sure we’re necessarily supported and encouraged to do joint research” (P13), which reflected that “further up the organisation….[research] might not be quite as high priority” (P23). “Senior leadership…buy-in” (P9) was considered paramount as it “sends a message…from the top…that they want to be part of co-production and they want to become more research minded” (P39). There was a common perception that “any drive towards cooperation will be at Research Director level” (P20), perhaps with “Board-level Research Directors” (P22), to educate and advocate “around the value [of collaboration]” (P23). Many organisations were considered “fully on board” (P18) and “totally committed to collaborative research” (P27); it is not “tokenistic” (P33).

There was reference to the collective responsibility and group identity in true collaborations: “if one partner fails, we’re all failing….if we’re packaging ourselves as a collaborative, that’s where we need to come in and support each other…it’s not just thinking about your own staff and your own interests needs” (P23), but having the “ability and the willingness to bring others along with us…partners who are…willing but don’t have the capacity” (P29). One stakeholder explained the benefits of group identity as a motivator to collaborate:

*if you’re working together as powerful organisations, then you’re going to get bigger studies…you then become a centre of excellence yourself…because you’re attracting bigger studies, better studies, you have a great deal more choice of expertise to be in the teams for those studies, you’ve got the pick of the best* (P17).

Overall, there was a belief that collaborating organisations needed to have “a degree of harmony and common vision, but also everybody needs to be hitting their institutional targets” (P22). This will involve “understanding where everyone is coming from” (P10).

#### TDF domain: beliefs about capabilities

Some organisations clearly lacked the confidence to try something new and were not “confident being the first to do something…[and] looking to see if another Council somewhere else in the country has tried something similar” (P13). Others expressed organisational fear of “bad press…a fear of blame consequence, reputation…that definitely hinders innovation as well because people are scared of failing” (P10).

Many stakeholders were more confident in their ability to “deliver high quality research” (P29), reported having “great expertise in the delivery side [of research]” (P23), or even that they were “pure research…we are all ready and we’re all experts in what we do” (P29). This expertise and activity was recognised as important for developing a track record and then building relationships that encourage other organisations into collaborations: “we do have credibility in the outside world, which…brings the external collaborators to us” (P15).

#### TDF domain: beliefs about consequences

In addition to beliefs about the beneficial consequences of collaborative R&I (e.g., skills- and capacity-sharing), collaboration was considered necessary to address the needs of “a big geographical area…if we don’t work together, we’re not going to get the kind of achievements that we need” (P4) and given the shift towards integrated care:

*our service has become more integrated…research will have to follow that, because you won’t be able to…write a protocol that’s designed for a service that runs in silo, because that won’t exist* (P5).

Others noted potential risks of collaboration, often based on negative experiences of imbalanced partnerships. For example, one stakeholder described how they had “tried to collaborate…but…[the other organisation] didn’t want to…because they want(ed) to keep all their research activity to themselves” (P26). Other collaborations were unsuccessful as “one partner wanted to come in and take over” (P23), with situations described as “not necessarily a partnership, it’s perhaps a dictatorial relationship” (P9). Those in smaller organisations feared that their “voice can be lost” (P21) which created “concerns about being swallowed up by some of the bigger [organisations]” (P5). One stakeholder from a large organisation explained that “trying to maintain engagement from partners…has been quite difficult…we want to drive it forward, but then…we end up owning it rather than it being more collegiate…because of capacity” (P10). Several stakeholders were keen for agreements to ensure equitable distribution of workload:

*there has to be an agreement between parties that they are actually not going to just leave it to one organisation to do all the work. There has to be sharing of the workload…otherwise…it’ll cause resentment and then that affects future partnerships* (P17).

This imbalance also had financial implications that might further limit motivation to collaborate:

*if the accrual all goes to the originating partner organisation, and the money stays in there, then that’s going to make an already challenging financial situation…even worse…the work required is way beyond the funding support available for it and you know that you’re just digging yourself a financial grave if you take part (P20)*.

To mitigate such negative consequences and build motivation for collaboration, all partners should “have an equal voice within the collaboration” (P21), whereby “smaller partners at the table… are listened to” (P9). There also “has to be trust that everybody’s going to get the right acknowledgement for their input” (P17), which also extended to appropriate resource allocation among partners: “if you’re involved in research, which has costs, you need to make sure that the benefits get distributed pro-rata between the participating organisations” (P20).

#### TDF domain: goals

Goals for collaborative R&I at organisational level ranged from improving the “R&I footprint at the Board” (P22), “showing that collaboration has material benefits” (P20), being “more structured in terms of how we approach innovation” (P13), developing “homegrown research…rather than mainly focusing on commercial work” (P22), and to “increase…collaborations over the next five years” (P17).

Specific actions to support these goals and overcome some of the aforementioned barriers often involved the wider system, although were caveated with the risk that individuals lack “the time or the headspace” (P26) for such innovative activities. First, creation of a shared framework to support organisations in R&I, whereby “all the individual organisations have had some input into that framework, there’s more chance of it being successful” (P18). This engagement could avoid reticence through organisations believing “a universal framework across the system might undermine or require them to change in some way” (P5).

Second, stakeholders discussed other practical ways to improve inter-organisational communication and relationships, such as a staff “directory of who is who” (P20):

*it’s a win, win, situation for everybody…if we held a registry, and we knew what everybody did…we could make those collaborations really easily…we could attract much…bigger research [and] we could bring more funding, more resources into the region (P17)*.

Other suggested initiatives were “seminars and workshops, actually worked really well at getting people together and chatting” (P4). In turn, stakeholders can become familiar with “where expertise is…[which] leads on to the next project…and then you start to get really cohesive teams working together” (P4). Ultimately, stakeholders wanted to reach “a point where we need to formalise the communication channel” (P20) by “having one main point of contact…and then those people facilitate the rest of the contacts” (P10).

Third, to address skill and capacity needs, there was a perceived need to think “creatively about how we…[allow] staff to move more fluidly throughout our organisations” (P23); for example, having “people on contracts that are allowed to work in other trusts, ability to second people, ability to borrow statisticians, or other people that have got specialist expertise” (P32). In addition, “we have to make…[research] somebody’s role and not add it on to their existing job” (P9) which would create “more capacity…[to] go out…in person and try and get people on board and interested in taking part in research” (P26).

#### TDF domain: optimism

There was optimism among stakeholders regarding the prominent future role for “research, development, and innovation and in equal quantities” (P33), which would be more successful with collaboration: “greater depth in terms of research, governance, and capacity, if we work together more collaboratively” (P22). Again, stakeholders noted the potential benefits of organisations with “different strengths and different weaknesses…able to complement each other” (P18), and attracting “much larger studies…to this region…because you’re a stronger team” (P17). For smaller or less research-active organisations, this could give them “much more…power trying to get different studies because…[larger organisations] can take part in studies and then we can tag on to the back of them” (P26), which had additional benefits as it “shares the risk, it shares a load” (P21).

Collaboration was seen as a way to “get better quality research…[and] there’s enough evidence out there to show…it’s absolutely critical” (P4), and to generate innovation:

*more innovative approach to research…by just through that the sharing of good practice and sharing of good ideas…people just becoming more aware of research opportunities and how they can integrate research into their everyday jobs without it being completely onerous* (P25).

Stakeholders also expressed optimism around the benefits of collaborative R&I in helping to improve care, helping to “break new grounds, provide better forms of treatment” (P35):

*there’ll be huge benefits particularly for our people that we work with…everybody is enthusiastic, everyone is committed, it’s just a work in progress to embed those things…[and] if we’re just more joined up as a whole, it can only serve to provide a better service* (P39).

Stakeholder pessimism related to aforementioned barriers. There were doubts about the integrity of collaborations when involving other organisations, which again signals the need to overcome a competitive mindset: “if I come up with a novel idea…there is a risk of someone else…pinching my idea” (P15). True collaboration could be limited by the perception (or reality) that some organisations will “be out to get what they can get and not want to give back” (P23). Lack of capacity among clinicians was noted: “it is easy when you are an academic institution, or researchers who are funded full time, to wonder why your clinical partners are not pulling their weight” (P22), particularly when adding the “requirements of collaboration just means that something else has to be dropped” (P20).

## Discussion

### Main findings

Stakeholder interviews indicated that, in general, organisations were ***motivated*** to undertake R&I and wanted to collaborate with ICS partners. They recognised the potential benefits to their organisation (e.g., skill-sharing, staff development, attracting larger studies and funding), and the wider system, in terms of needing to integrate research alongside integrated care, and potential benefit for care recipients. There was related optimism, around building strengths, capacity and skills for research, but also some barriers that could undermine motivation, such as experiences of unsuccessful or imbalanced partnerships, fear of failing and lack of confidence within less research active organisations. More fundamental barriers were evident as limitations on ***opportunities*** for collaborative R&I, which were largely through influences at organisation or system levels. Many stakeholders spoke of an historical culture of competition, particularly around research and related funding that remained an impediment to true collaboration, and a lack of research culture and prioritisation, which was made more difficult in the current economic climate. Other barriers related to ***capability***, with marked inter-organisational variation and imbalance in research skills (at individual and organisational levels), and related capabilities and confidence for R&I. This reflected the range of partners, from local authorities (who often commission, rather than undertake research/evaluation), to large NHS Trusts with extensive research portfolios, and academic institutions.

Our findings resonate with a wider literature on inter-agency collaboration between health and non-health care organisations. A 2022 meta review identified many common issues: the need for frequent communication and sharing of information and best practice to build trusting relationships; competing organisational agendas that undermine partnerships; the importance of shared processes and systems (e.g., data sharing agreements), joint meetings, and planning processes [25]. There are also specific issues to consider for collaborations between organisations that differ in their research culture/experience and/or belong in different sectors (e.g., differences in timescales, thinking and priorities, perceived financial costs of academic involvement, and not knowing who to connect with) [26].

Across stakeholders from the range of ICS partner organisations, we observed a perceived need to change the mindset, to prioritise R&I, and shift from competition to collaboration. Stakeholders in this study and elsewhere, have noted the need for the buy-in of senior leaders or those at ‘board-level’ to affect such change. Senior leaders are central. There is a recognised need for more collaborative leadership to promote working across organisational and professional boundaries [27], while influencing motivation, helping to agree common aims, shaping local collaborations, and, critically, to free up necessary resources [25]. This has been highlighted in the social care context, where organisations that historically lack a strong research culture (e.g., local authorities) need senior leaders to help embed research as an integral part of their organisation’s role [28, 29]. In such circumstances, research activity might survive, despite challenges from the wider system, such as national policy, institutional or sector context, political context, or as noted here, the social and economic context [25]. It was clear for our stakeholders that allowing the time and resource for research, or trying to change practice to be more collaborative, was made particularly challenging at a time of under-funded and over-stretched public services. Here, collaboration should be viewed as part of the solution, given the potential value of resource sharing across agencies as a mechanism to facilitate joint working [25, 30].

The entrenched culture of competition was often cited by stakeholders, primarily in the context of research. However, competition is a wider and prevailing way of working for many ICS partners. Health and care providers have operated within a variously competitive funding environments over the last decade [31]. There is debate about the relative merits of collaboration versus competition for healthcare, and the balance has changed. The Social Care Act 2012 (Lansley Reforms) requirements for competition and competitive tendering, were quickly followed in 2014, by regulators mandating some form of collaboration [31]. More recently, the NHS Long Term Plan advocated for greater collaboration between primary and secondary care, between Trusts, and across providers [1]. Voluntary and community sector providers often compete against one another to offer similar services in local competitive tendering processes [32], and Universities routinely compete for limited research funding [33]. Therefore, moving from competition to collaboration for R&I will require deliberate and sustained changes in approach and with supportive processes, but such a moves does align with ICS development (and reflect research-related ICB duties [9]). As the King’s Fund report noted in the context of collaboration across health and care:

*Effective working across organisations means adopting new practices to navigate challenges such as conflicting organisational goals, competing institutional norms and rules, and any perceived loss of power or resource* [27, p2].

Stakeholders in the present study proposed some specific actions to support the drive for collaboration, some at ICS or system level, and others at the level of individual organisations (Table 2). When aligning these with the corresponding COM-B policy or intervention types [14], most actions related to education or training, trying to change the environment (social) to be more conducive to collaborative research and creating the capacity for collaboration. Those at the ICS level fall under the remit of the SSHERPa network, and highlight the need for leadership by larger, more research-active organisations able to coordinate, share skills and provide training, whilst being sensitive to power imbalances and the role of smaller partners.

**Table 2:** Summary of recommendations to support collaborative working

Aim	Possible COM-B policy (P) or intervention (I) types [definition from (14)]	Organisational Level	ICS/system level
Share skills and knowledge (Capability)	Education (I)– increase knowledge or understanding Environmental restructuring (I)– change physical or social context Training (I)– impart skills Modelling (I)– provide example for people to aspire to or imitate	Executive Board Level responsibility for research	Process development/ mapping to share knowledge/skills Webinars/events, training Shared roles working across organisations (shadowing/induction)
Reduce competition (Opportunity)	Education (I)– increase knowledge or understanding Persuasion (I)– use communication to induce positive or negative feelings or stimulate action Incentivisation (I) - creating expectation of reward	Board level commitment to partnership working for research	Develop common aims– shared framework Transparency over funding streams
Developing skills, capacity and networking (Capability)	Environmental restructuring (I) - changing physical or social context Environmental/social planning (P) - design and/or control the physical or social environment Training– impart skills Education– increase knowledge or understanding	Job planning/ protected time for research– built into recruitment and retention strategies	Directory of staff and services– who’s who Webinars/events, training One point for contact– to help facilitate introductions
Building trust and reducing risk (Capability, Motivation)	Guidelines (P) - create documents that recommend or mandate practice Regulation (I)- Establishing rules or principles of behaviour or practice Environmental restructuring (I)- changing physical or social context		Data sharing agreements Collaboration agreements Directory of who’s who to build relationships One point for contact– to share opportunities

Critically, ICS development intends to effect major systems change. Such change requires partners to coalesce around long-term process-orientated goals that can shift behaviours in the desired direction. Part of this should be ‘*encouraging stakeholders from different parts of the system to work together with the aim of aligning goals, resources and activities*’ [34, p9]. The same can be applied to the shift to truly collaborative R&I. Some of the goals or actions suggested by stakeholders can serve that shift and help to develop relationships across organisations (e.g., Board-level representation for R&I, shared directory, shared staff), rather than focusing on outcome goals (e.g., to undertake more collaborative R&I). Ultimately, this comes down to long-term investment in organisational relationships, and creating the conditions for true partnership and collaborative working.

*We need to identify and create the incentives and levers in the system to ensure we enable different ways of working– both in individual organisations and collaboratively across the system* [35, p17].

#### Strengths and limitations

Strengths of this study included the large qualitative sample, range of stakeholders and represented organisations. Application of well-established frameworks was intended to understand the individual and organisational influences on collaboration across the ICS, with the COM-B model enabling translation of findings into potential actions to promote collaborative R&I. However, much the data collected at individual level (albeit from individuals with organisational influence) related to *organisational* behaviours and covered influences at the organisational level. Therefore, rather than relying on the TDF, alternative frameworks developed specifically to understand organisational behaviours could have provided greater insight. For example, the Consolidated Framework For Implementation Research (CFIR) [36] could have been adapted for use alone or in combination with the TDF [37]. Other models of cooperation might also have been appropriate (e.g. Bergen Model of Collaborative Functioning (BMCF) [38]; Diagnosis of Sustainable Collaboration (DISC) model [39]). Other limitations were the lack of voluntary and community sector organisation representation, and inclusion of stakeholders from only two ICSs.

## Conclusion

Overall, qualitative data from stakeholders across ICS partners identified barriers that seriously limited their opportunities for collaborative R&I; specifically, an organisational culture in which research was not prioritised or was competitive (not collaborative), compounded by financial pressure and excessive demand in the wider system that prevented investment in R&I. In this context, changes that require additional investment or activities seem unrealistic. Despite these challenges, motivation within organisations for collaborative R&I remained high, as it presents as a vehicle and opportunity for some of these issues to be addressed and collaborative working to be supported. In turn, barriers around capability or factors undermining motivation would likely diminish. Using the COM-B, some types of intervention or policy change are suggested to influence the behaviour of organisations (at or wider ICS level). This could be further explored using frameworks more specific to organisational behaviour, and through taking a systems change approach to help create an environment for collaborative R&I alongside the overarching systems change towards integrated care.

### Electronic supplementary material

Below is the link to the electronic supplementary material.

Supplementary Material 1

## Data Availability

All data generated and analysed during the current study are not publicly available due to the confidential nature of participant transcript data.
