# Supplementary material for: “We have to change our mindsets”: a qualitative study of barriers and facilitators in research collaboration across integrated care system organisations
Source: BMC Health Serv Res. 2024 Mar 1;24:264. doi: 10.1186/s12913-024-10760-3 (PMC10908113; doi:10.1186/s12913-024-10760-3)
Supplement: Supplementary file 1 — Supplementary Material 1 [file 12913_2024_10760_MOESM1_ESM.pdf]

Interview Topic Guide

1. What type of organisation do you work for?
   NHS/Local authority/Third sector/Other

2. Please can you tell me a bit about your job
   a. What is your job title?
   b. How long have you worked there?
   c. What is the nature of your day-to-day role?
   d. How is does your role within your organisation relate to research and/or innovation?

3. Do you consider your organisation to be **research active**?
   Why do you say that/please can you explain?

4. How involved in **innovation** do you consider your organisation to be?
   Why do you say that/please can you explain?

5. Do you think collaborative research and innovation between organisations in the Integrated Care Systems (ICSs) will be important?
   Please can you explain why you think that?
   What would be the benefits?
   Any risks/disbenefits/negative consequences?

6. Knowledge and skills
   a. Are you aware of the processes and procedures for conducting R&I in your organisation?
   b. Are there any additional processes or procedures for R&I that involves collaborating with other organisations?
   c. What skills (if any) do you have, personally or exist within your organisation, that could support collaborative R&I?
   d. Are there any gaps or areas that you would like to improve, or that your organisation needs to address?

7. The next few questions relate to collaboration between organisations around research and innovation in Staffordshire and Stoke-on-Trent / Shropshire, Telford and Wrekin:
   a. To what extent do you feel that your organisation is committed to collaborative R&I? why do you say that? please can you explain why?
   b. To what extent is collaborative R&I encouraged and supported in your organisation?
   c. What factors support or hinder you in collaborating with other organisations on R&I?
   d. How well do systems in place support you in collaborating with other organisations on R&I?
   e. Can you think of any examples of successful collaborative R&I?
   f. Can you think of any examples where this has been tried, but not been successful?
   g. If research or innovation is part of your role, do you feel supported in this?

8. Are there any other barriers to collaborating with other organisations for R&I that we have not discussed so far?
   What are they?
   Can you think of any examples?

How might these be resolved?

9. In what ways (if any) would your organisation benefit from collaborating with others on research or innovation?

10. If you do not think that a joint approach is feasible or beneficial, please can you explain your reasons?

11. Do you have ideas on how we could further strengthen and support collaborative working across the system?

12. What are the main barriers to us developing an integrated research and innovation framework for Staffordshire and SoT/Shropshire, Telford and Wrekin?
    Please give examples if possible

13. Is there anything else you wanted to say on this topic that we have not covered?

Thank you very much for your time
